# Type 1 diabetes genetic risk score is discriminative of diabetes in non-Europeans: evidence from a study in India

**DOI:** 10.1038/s41598-020-65317-1

**Published:** 2020-06-11

**Authors:** James W. Harrison, Divya Sri Priyanka Tallapragada, Alma Baptist, Seth A. Sharp, Seema Bhaskar, Kalpana S. Jog, Kashyap A. Patel, Michael N. Weedon, Giriraj R. Chandak, Chittaranjan S. Yajnik, Richard A. Oram

**Affiliations:** 1 Institute of Biomedical and Clinical Science, University of Exeter Medical School, Exeter, Devon, UK; 2 Genomic Research on Complex diseases (GRC Group), CSIR-Centre for Cellular and Molecular Biology (CSIR-CCMB), Uppal Road, Hyderabad, 500 007 India; 3 KEM Hospital, 489 Rasta Peth, Sardar Moodaliar Road, Pune, 411011 India; 4 National Institute for Health Research Exeter, Clinical Research Facility, Exeter, UK

**Keywords:** Type 1 diabetes, Genetics research, Molecular medicine, Risk factors

## Abstract

Type 1 diabetes (T1D) is a significant problem in Indians and misclassification of T1D and type 2 diabetes (T2D) is a particular problem in young adults in this population due to the high prevalence of early onset T2D at lower BMI. We have previously shown a genetic risk score (GRS) can be used to discriminate T1D from T2D in Europeans. We aimed to test the ability of a T1D GRS to discriminate T1D from T2D and controls in Indians. We studied subjects from Pune, India of Indo-European ancestry; T1D (n = 262 clinically defined, 200 autoantibody positive), T2D (n = 345) and controls (n = 324). We used the 9 SNP T1D GRS generated in Europeans and assessed its ability to discriminate T1D from T2D and controls in Indians. We compared Indians with Europeans from the Wellcome Trust Case Control Consortium study; T1D (n = 1963), T2D (n = 1924) and controls (n = 2938). The T1D GRS was discriminative of T1D from T2D in Indians but slightly less than in Europeans (ROC AUC 0.84 v 0.87, p < 0.0001). HLA SNPs contributed the majority of the discriminative power in Indians. A T1D GRS using SNPs defined in Europeans is discriminative of T1D from T2D and controls in Indians. As with Europeans, the T1D GRS may be useful for classifying diabetes in Indians.

## Introduction

As of 2015 there were 490,000 children <15 years of age living with type 1 diabetes (T1D) globally, with >100,000 in India^1^. Despite this, almost all large genetic, biomarker and phenotype studies focus on T1D in people of European ancestry. The extent of overlapping genetic risk for T1D between different ethnic populations is not well described. Rising obesity rates and the recognition that T1D can occur at any age make the discrimination between T1D and type 2 diabetes (T2D) an increasingly difficult challenge. The discrimination of diabetes subtypes is more challenging in the Indian population due to the higher prevalence of early-onset T2D at a lower body mass index (BMI) than in European populations^2^. It is vital to identify the correct diabetes subtype as optimal treatment differs between T1D and T2D.

We and others have previously shown that a T1D genetic risk score (T1D GRS) comprising of between 9 and 67 SNPs can be a useful tool to aid the discrimination between T1D and T2D or controls in Europeans^3–6^. The majority of the discriminative power of the T1D GRS is in the first 9 SNPs when ranked by effect size^3^. These 9 SNPS include SNPs tagging the high-risk HLA DR3-DQ2.5 (DR3)/DR4-DQ8 (DR4) alleles and the highly protective HLA DR15-DQ6.2 (DR15) allele. In this study we aimed to assess if the T1D GRS developed in Europeans discriminates T1D from T2D and controls in Indians and could aid diabetes classification in this population.

## Methods

In order to assess the performance of the T1D GRS we studied a cohort from Pune, India of 305 people with T1D, 352 people with T2D and 334 people without diabetes (Table 1). For comparison to Europeans we used the Wellcome Trust Case Control Consortium (WTCCC) study comprising of 1963 people with T1D, 1924 people with T2D and 2938 people without diabetes^7^.

**Table 1:** Characteristics of Indian cohort. Data presented as median (25^th^–75^th^ Centile) unless otherwise stated. T1D aab positive indicates positivity for any one of 3 antibodies (GAD, IA2, ZnT8) tested. NA: Not applicable; ND: Not Done.

Cohort	T1D aab positive	T1D (clinically defined)	T2D	Controls
N	200	262	345	324
Males (%)	42	40.5	41.7	53.1
Age at diagnosis (years)	10.0 (6.9–13.4)	9.8 (6.2–13.2)	51.0 (47.3–55.5)	NA
Age at sample collection (years)	13.9 (9.5–19.6)	14.6(9.9–20.9)	56.3 (51.8–61.6)	31.4 (27.5–35.3)
Duration of diabetes (years)	2.9 (0.1–7.2)	3.6 (0.3–8.4)	9.3 (7.0–12.7)	NA
BMI Kg/m^2^	XX	17.4 (15.0–19.9)	28.0 (26.3–29.6)	19.4 (17.6–22.0)
At least one Islet autoantibody [n(%)]	N/A	200(76%)	ND	ND
Random C-peptide (pmol/L)	29.0 (<3–144.7)	22 (<3–128)	ND	ND

## Cohort characteristics

### Indian cohort

We studied individuals with and without diabetes from studies in Pune, in the state of Maharashtra, India, who were of self-reported Indo-European ancestry.

#### T1D

We considered people with T1D eligible for the study if they were attending the outpatients clinic of the Diabetes Unit, King Edward Memorial Hospital and Research Centre, Pune. We defined T1D as diabetes diagnosed <30 years of age, on insulin treatment from diagnosis and with a history of ketoacidosis. We excluded any sample which failed genotyping QC (n = 4). To reduce the risk of including people with monogenic diabetes or T2D we also excluded people diagnosed at age <9 months if islet autoantibody negative and those with random serum C-peptide concentrations >600 pmol/L who were islet autoantibody negative^8^ or who had missing data in any of the measures. 262 people met the inclusion criteria and had clinically defined T1D. We performed analyses of this group and of a group defined by a stricter definition of T1D that included only autoantibody positive individuals (n = 200) in case some autoantibody negative individuals had non-T1D.

#### T2D

People with T2D (n = 352) were recruited as part of the Wellcome Genetic collection (WellGen) of people with diabetes mellitus^9^. For this study we selected people with T2D who were diagnosed >45 years and were on oral anti-diabetic agents for more than 5 years after diagnosis. We excluded those clinically judged to have exocrine pancreatic disease, monogenic diabetes or insulin-dependence (history of ketoacidosis, unresponsiveness to oral hypoglycaemic agents, on continuous insulin treatment since diagnosis). We excluded any samples which failed genotyping QC (n = 7), leaving 345 people with T2D in final analysis.

#### Control subjects

Control subjects (n = 334) were people without diabetes (75 g oral glucose tolerance test; WHO 1999 criteria) residing in and around Pune, India. These included parents of children from the Pune Children Study^10^ - a study on the relationship between child’s birthweight and future risk for T2D. Ten samples did not pass genotype QC leaving 324 controls.

Informed consent was obtained from all study participants who were above 18 years of age. For those below 18 years of age informed consent was obtained from a parent and/or legal guardian and for those between the ages of 12 to 18 years an additional consent was obtained from the participant.

The collection of clinical data and use of biobanked samples for the biochemical, immunological and genetic measurements was sanctioned by the Institutional Ethics Committee of the KEM Hospital Research Centre, Pune, India (KEMHRC ID No1737 & KEMHRC ID No PhD19) and all methods were performed in accordance with the relevant guidelines and regulations.

### WTCCC Cohort

To compare the results generated in our Indian cohort to results from Europeans we used the WTCCC cohort described previously^7^:

#### T1D

The WTCCC T1D cohort (n = 1,938) all received a clinical diagnosis of T1D at <17 years of age and were treated with insulin from the time of diagnosis.

#### T2D

The WTCCC T2D cohort (n = 1,914) were all diagnosed between 25 and 75 years of age, were Glutamic Acid Decarboxylase (GAD) autoantibody negative and were either treated with diet/oral hypoglycaemic agents or had an interval of at least 1 year between diagnosis and the institution of insulin therapy.

#### Control subjects

The WTCCC control subjects (n = 2938) were made up of people with no known diagnosis of diabetes from two sources: the 1958 British Birth Cohort and the UK Blood Services control cohort.

### Genotyping

We genotyped 9 SNPs that capture the majority of discriminative power of the T1D GRS developed using subjects of European ancestry from the WTCCC study^7^ (Table S1). Genomic DNA was isolated from all samples using the salt precipitation method and DNA samples were plated in 96-deep-well storage plates at a uniform concentration of 10 ng/λ at the CSIR-Centre for Cellular and Molecular Biology, Hyderabad, India. Each plate includes eight repeat samples (∼10%) as a quality control measure. We used the Sequenom Mass Array technology to genotype 9 SNPs as a part of multiplex pool. We had >98% genotype success rate and >97% concordance between results of duplicate samples.

### Autoantibody and C-peptide measurement

We measured random serum C-peptide concentration by direct electrochemiluminescence immunoassay (Cobas C-peptide kit, Roche Diagnostics GmBH, Germany; lower detection limit: 3.3pmol/l with CV of 0.6% at 33 pmol/L) on a Cobas e411 analyser^11^.

We measured GAD autoantibody, Insulinoma antigen 2 (IA2) antibody and Zinc transporter 8 (ZnT8) antibody by using a commercial ELISA kit (RSR Limited, Cardiff, UK). Individuals were considered positive for antibodies if their results were GAD > 10 IU/ml, IA2 > 7.5 IU/ml and ZnT8 > 15 IU/ml.

### GRS calculation

The T1D GRS was calculated by:

For the non-DR3/DR4 SNPs - summing the dosages of the risk increasing allele at each locus (between 0 and 2) multiplied by the weight (ln(odds ratio[OR])) for each of the 7 SNPs (Supplementary table S1). For DR3/DR4-DQ8 contribution - the DR3/DR4-DQ8 haplotypes were imputed and the corresponding weights assigned to each individuals score^12^ (as in^13^). (Supplementary table S1). The sum of these two was then divided by 15. This assumes that the score follows a log-additive model for T1D risk.

For analysis of discriminative power of HLA alone we created 4 groups defined by imputed haplotypes: low risk (category 0) with no DR3 or DR4, medium risk (category 1) with a single copy of DR3 or DR4, high risk (category 2) with two copies of DR3 or DR4, very high risk (category 3) with one copy of DR3 and one copy of DR4. This was used as an ordinal variable in logistic regression.

### Statistics

We tested the ability of a 9 SNP T1D GRS to discriminate between T1D and T2D and controls by using the area under the curve (AUC) of the receiver operator characteristic (ROC) statistic. All analyses were carried out using PLINK v1.90^14,15^ and STATA 15(StataCorp LP, College Station, TX). Comparisons of discrimination were made using the roccomp package.

## Results

### T1D GRS is discriminative of T1D from T2D in an Indian clinic

**Figure 1:**
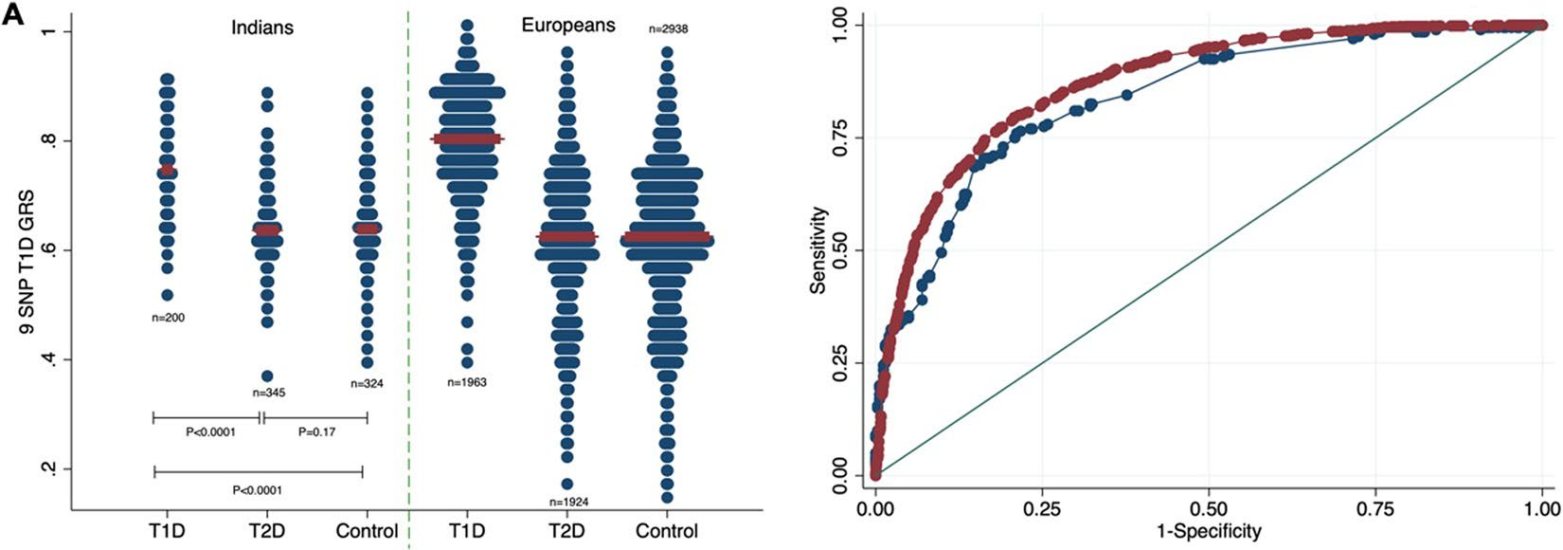
Comparison of the ability of a 9 SNP GRS to discriminate between T1D and T2D in Indians and Europeans. (**A**) Dotplot of 9 SNP T1D GRS in T1D, T2D and controls in Indians and Europeans. The width of the blue bars indicates frequency and the red line indicates the median. (**B**) ROC curves showing the power of the 9 SNP T1D GRS to discriminate between T1D and T2D in Indians (blue: AUC [95% CI] 0.84 [0.80–0.87]) and Europeans (red: AUC [95% CI] 0.87 [0.86–0.88]), *P* < 0.001.

In Indians, the 9 SNP T1D GRS was higher in people with autoantibody positive T1D (n = 200) than in people with clinical T1D but who were autoantibody negative (n = 62) (mean [SD] T1D GRS was 0.76[0.09] v 0.73[0.1], p = 0.03). This suggested that there may be some cases of misclassified T1D in the clinically defined cohort, so we proceeded using only those with clinical T1D and a positive autoantibody for the remainder of the analysis. The T1D GRS was considerably higher in T1D compared to T2D or controls (T1D vs T2D (mean [SD]) 0.76 [0.09] vs 0.64 [0.07], *P* < 0.0001; T1D vs controls 0.76 [0.09] vs 0.65 [0.07], *P* < 0.0001). The 9 SNP T1D GRS for the Indian people with T1D was lower than the European people with T1D (mean [SD]) 0.76 [0.09] vs 0.80 [0.09] respectively, *P* < 0.0001) (Fig. 1A, Supplementary table 3). However, the 9 SNP T1D GRS was still strongly discriminative of people with T1D from people with T2D (ROC AUC [95% CI] 0.84 [0.80–0.87]) (Fig. 1B) and people with T1D from controls (0.82 [0.78–0.85]) (Supplementary figure 1) in the Indian cohort. The power of the 9 SNP GRS to discriminate people with T1D from people with T2D was slightly lower in Indians compared to Europeans (ROC AUC [95% CI] 0.84 [0.80–0.87] vs 0.87 [0.86–0.88] respectively, *P* < 0.0001, Fig. 1B).

### HLA DR3/DR4 status is the major discriminator of T1D

The discriminative power of imputed HLA DR3/4 typing alone accounted for the majority of the power of the T1D GRS to discriminate people with T1D from people with T2D and controls in both Indians and Europeans and was similar between Indians and Europeans. As with the 9 SNP GRS the discriminative power was lower in Indians compared to Europeans (T1D v controls ROC AUC [95% CI] 0.79 [0.75–0.83] vs 0.82 [0.81–0.83], *P* = 0.16) (Supplementary figure 2 & Supplementary figure 3).

### Odds ratios and risk allele frequencies at DR3 and DR4 are different in Indians compared to Europeans

**Figure 2:**
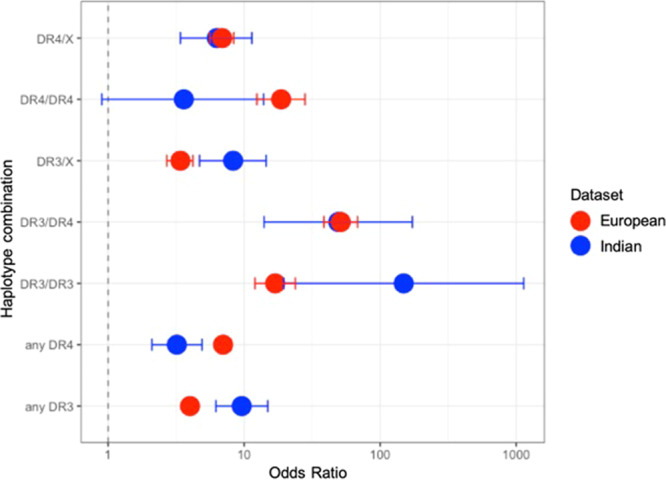
Comparison of odds ratios for DR3/DR4 haplotype combinations between Indians (blue circle) and Europeans (red circle). Bars show 95% confidence intervals.

We assessed the individual odds ratios for each of the variants contributing to the 9 SNP GRS. This showed the DR3 and DR4 combinations to be different between Europeans and Indians (Fig. 2, Table 2 and Supplementary figure 4). The presence of either of DR4 and DR3 increased the odds ratio of T1D in the Indian cohort, however the strength of effect was reversed compared to Europeans. The odds ratio for T1D in Indians with DR4 was lower (OR [95% CI] for any DR4: 3.2 [2.1–4.9] in the Indian cohort vs 7.0 [6.2–8.0] in Europeans) and the odds ratio for T1D in Indians with DR3 was higher compared to Europeans (OR [95% CI] for any DR3: 9.6[6.2–14.9] in the Indian cohort vs 4.0[3.6–4.5] in Europeans).

**Table 2:** Comparison of odds ratios and allele frequencies between Indians and Europeans. 95% confidence intervals shown in brackets. *P* value for comparison of odds ratios.

	Indians			Europeans			
	OR	T1D	CONT	OR	T1D	CONT	*P*
DR3/DR4	49.2	0.14	0.01	51.3	0.34	0.03	0.95
	14.0–172.2)			(38.6–68.1)			
DR4/DR4	3.6	0.02	0.02	18.7	0.06	0.01	0.02
	(0.9–13.9)			(12.4–28.0)			
DR3/DR3	148.8	0.27	0	16.9	0.09	0.02	0.04
	(19.5–1136.2)			(12.0–23.8)			
DR4/X	6.3	0.18	0.11	6.9	0.26	0.17	0.79
	(3.4–11.4)			(5.6–8.4)			
DR3/X	8.3	0.27	0.10	3.4	0.15	0.21	0.004
	(4.7–14.5)			(2.7–4.2)			
any DR3	9.6	0.56	0.12	4	0.58	0.26	0.0001
	(6.2–14.9)			(3.6–4.5)			
any DR4	3.2	0.34	0.14	7	0.66	0.21	0.0005
	(2.1–4.9)			(6.2–8.0)			
rs3129889 (HLA-DRB1*15)	0	1	0.99	9.1	0.99	0.86	XX
	(0-0.76)			(6.4–13.0)			
rs2395029 (HLA-B*5701)	1.1	0.98	0.96	2.9	0.99	0.96	0.07
	(0.4–2.9)			(1.9–4.2)			
rs2476601 (PTPN22)	1.8	0.02	0.01	2.1	0.17	0.1	0.79
	(0.5–6.4)			(1.8–2.5)			
rs689 (INS)	2.8	0.94	0.86	1.6	0.80	0.7	0.72
	(1.6–5.1)			(1.4–1.8)			
rs12722495 (IL2RA)	1.3	0.95	0.95	1.5	0.92	0.89	0.61
	(0.6–2.5)			(1.25–1.8)			
rs1264813 (HLA-A*24)	1.4	0.19	0.17	1.6	0.13	0.1	0.62
	(0.9–2.1)			(1.3–1.9)			
rs2292239 (ERBB3)	1.5	0.31	0.24	1.4	0.40	0.34	0.7
	(1.0–2.1)			(1.2–1.5)			

The frequencies of major risk alleles DR3 and DR4 were lower in the Indian controls compared to European controls (Indian controls: any DR3 12% frequency, any DR4: 14%; European controls: any DR3 26%, any DR4 21%). The major protective HLA allele in Europeans is DR15-DQ6. As expected this had a very low frequency in both Indians and Europeans (Table 2). The DR15-DQ6 allele had a much lower frequency in Indian controls (1%) than European controls (14%) (Table 2). This finding is consistent with results from Indian individuals in the 1000 Genomes Project (2%)^16^.

## Discussion

In this study we demonstrate that a T1D GRS derived from Europeans can still be discriminative of T1D from T2D in Indians. The large numbers of people with T1D in India, the increasing recognition of childhood and early adulthood onset of T2D and recent studies highlighting the frequency of adult onset T1D^17,18^ emphasize that tools such as the T1D GRS will be needed to discriminate people with T1D from people with T2D in non-European populations.

The utility of polygenic risk scores for prediction and diagnosis of disease is an area of increasing interest^19,20^. The most effective polygenic risk scores are those derived from large genome wide association studies in independent datasets of heritable diseases. These studies have most commonly been performed in large datasets of people of European ancestry as is the case for T1D^20–22^. The effects of population stratification and ethnicity on genetic associations of disease may hinder the utility of polygenic risk scores in non-Europeans^23,24^. This is in part due to differences in underlying risk allele frequencies between populations. A good example in our study is the strongly protective HLA DR15-DQ6 allele which is common in Europeans but virtually absent in the Indian population we studied. This explains some of the differences in baseline T1D risk between Indians and Europeans. Despite these limitations of population stratification our study confirms that the major T1D risk alleles in Europeans are also key risk alleles in Indians and even in its current form (biased towards a European population) the T1D GRS is still strongly discriminative of T1D from T2D.

The effect of the DR3 allele on T1D risk in Indians is greater than in Europeans. Conversely, the DR4 association with T1D was less strong. These findings strengthen earlier findings by other smaller studies^25–27^ which hint at a stronger DR3 association in India. This could be explained by an interaction with the environment. It is possible that a specific pathogen or environmental risk factor that interacts with DR3 is more prevalent in the Indian population or environment compared to the European population and/or a common environmental exposure related to DR4 risk may be less common. Further exploration of this could provide important new insights into the environmental triggers that lead to T1D. The linkage disequilibrium between the tag SNPs (rs2187668, rs7454108) and the DR3 and DR4 alleles could be different across the different populations which could lower the efficiency of the SNPs to tag the haplotypes. However, the accuracy of the DR3/DR4 haplotype combination classification in the 1000 Genomes Project data set (http://www.internationalgenome.org/home) was 98.4% using the DR3/DR4 tag SNPs.

## Limitations

The population of India is very heterogenous and more data from across India are needed to properly assess how representative this study is of all Indian ethnicities. The subjects in the study were of Indo-European ethnicity and it will be interesting to test the observations on Dravidian people, another major ethnicity in India. We did not perform autoantibody testing in the T2D group and therefore could not actively exclude slowly evolving T1D in this group. However, we used clinical criteria designed to exclude misdiagnosed T1D (by excluding those on insulin within 5 years) and therefore make the assumption this will have a very low prevalence in the T2D group. If the T2D group contained large numbers of people with T1D this would only give a more conservative estimate of the utility of the T1D GRS and the similarity between the European and Indian results suggests this is unlikely. We used odds ratios derived from Europeans for the T1D GRS. We hypothesize that if key HLA risk allele frequencies are similar and these alleles confer similar risk then the T1D GRS is likely to be similarly discriminative. The use of large genome wide association studies to generate the weights in the T1D GRS means the odds ratios are precise for a European population. A natural next step is to try to define genetic relationships in a large Indian cohort with T1D to generate an Indian specific T1D GRS and to test expanded T1D GRSs that include more HLA alleles and recently associated non-HLA loci. However, a critical issue is the power required to do this and without large sample sizes it is possible that a GRS defined in a small (e.g. <1000 cases) cohort may not improve discrimination of T1D.

It is likely that a combined approach to classification of T1D that uses clinical features autoantibodies and genetic risk will be optimal for clinical diagnosis. Once larger cohorts with clinical and biomarker information are assembled it will be possible to test this approach.

## Conclusion

In conclusion, we show that a 9 SNP T1D GRS can be used to help classify diabetes in Indians. This study suggests the T1D GRS could be an effective tool to aid discrimination of T1D from T2D in Indians. Our study additionally highlights differences in OR of key risk alleles for T1D that warrant further investigation.

## Supplementary information

Supplementary Information.

## Data Availability

In order to access data generated or used in this study not contained in the manuscript please contact corresponding authors.
