# Supplementary material for: Type 1 diabetes genetic risk score is discriminative of diabetes in non-Europeans: evidence from a study in India
Source: Sci Rep. 2020 Jun 11;10:9450. doi: 10.1038/s41598-020-65317-1 (PMC7289794; doi:10.1038/s41598-020-65317-1)
Supplement: Supplementary file 1 — Supplementary Information. [file 41598_2020_65317_MOESM1_ESM.docx]

**Type 1 diabetes genetic risk score is discriminative of diabetes in non-Europeans: evidence from a study in India**

James W. Harrison^1*^, Divya Sri Priyanka Tallapragada^2*^, Alma Baptist^3^, Seth A. Sharp^1^, Seema Bhaskar^2^, Kalpana S. Jog^3^, Kashyap A. Patel^1,4^, Michael N. Weedon^1§^, Giriraj R. Chandak^2§#^, Chittaranjan S. Yajnik^3§#^, Richard A. Oram^1,4#^

^*^Joint first authorship

^§^Joint senior authorship

^#^Joint corresponding authorship

1) Institute of Biomedical and Clinical Science, University of Exeter Medical School, Exeter, Devon, UK

2) Genomic Research on Complex diseases (GRC Group), CSIR-Centre for Cellular and Molecular Biology (CSIR-CCMB), Uppal Road, Hyderabad, 500 007, India

3) KEM Hospital, 489 Rasta Peth, Sardar Moodaliar Road, Pune, 411011, India

4) National Institute for Health Research Exeter, Clinical Research Facility, Exeter, UK

**Supplementary Tables**

| SNP | Effect allele | Gene | OR | Weight |
| --- | --- | --- | --- | --- |
| rs2476601 | A | PTPN22 | 1.96 | 0.67 |
| rs1264813 | T | HLA-A*24 | 1.54 | 0.43 |
| rs2395029 | T | HLA-B*5701 | 2.5 | 0.92 |
| rs3129889 | A | HLA-DRB1*15 | 14.88 | 2.70 |
| rs12722495 | T | IL2RA | 1.58 | 0.46 |
| rs689 | T | INS | 1.75 | 0.56 |
| rs2292239 | T | ERBB3 | 1.35 | 0.30 |
| rs2187668 rs7454108 | T C | DR3/DR4-DQ8 | 48.18 | 3.87 |
|  |  | DR3/DR3 | 21.12 | 3.05 |
|  |  | DR4-DQ8/DR4-DQ8 | 21.98 | 3.09 |
|  |  | DR4-DQ8/X | 7.03 | 1.95 |
|  |  | DR3/X | 4.53 | 1.51 |

**Table S1.** SNPs genotyped and weights used for calculating GRS.

|  |  | Median | IQR |
| --- | --- | --- | --- |
| Indians |  |  |  |
|  | T1D | 0.75 | 0.69-0.82 |
|  | T2D | 0.64 | 0.61-0.68 |
|  | CONT | 0.64 | 0.61-0.68 |
| Europeans |  |  |  |
|  | T1D | 0.80 | 0.74-0.88 |
|  | T2D | 0.63 | 0.55-0.71 |
|  | CONT | 0.63 | 0.55-0.72 |

**Table S2.** 9 SNP GRS in T1D, T2D and controls in Indians and Europeans.

|  |  | Indians | | | Europeans | | |
| --- | --- | --- | --- | --- | --- | --- | --- |
|  |  | T1D | T2D | Controls | T1D | T2D | Controls |
| Indians | T1D |  | <1E-300 | <1E-300 | 1.00E-10 | <1E-300 | <1E-300 |
|  | T2D |  |  | 0.17 | 4.63E-125 | 0.028 | 0.04 |
|  | Controls |  |  |  | 2.61E-113 | 0.0016 | 0.002 |
| Europeans | T1D |  |  |  |  | <1E-300 | <1E-300 |
|  | T2D |  |  |  |  |  | 0.94 |
|  | Controls |  |  |  |  |  |  |
|  |  |  |  |  |  |  |  |
|  |  |  |  |  |  |  |  |
|  |  |  |  |  |  |  | sig |
|  |  |  |  |  |  |  | non sig |

**Table S3.** *P* values for Wilcoxon rank-sum test showing the difference between all groups.

**Supplementary Figures**

**
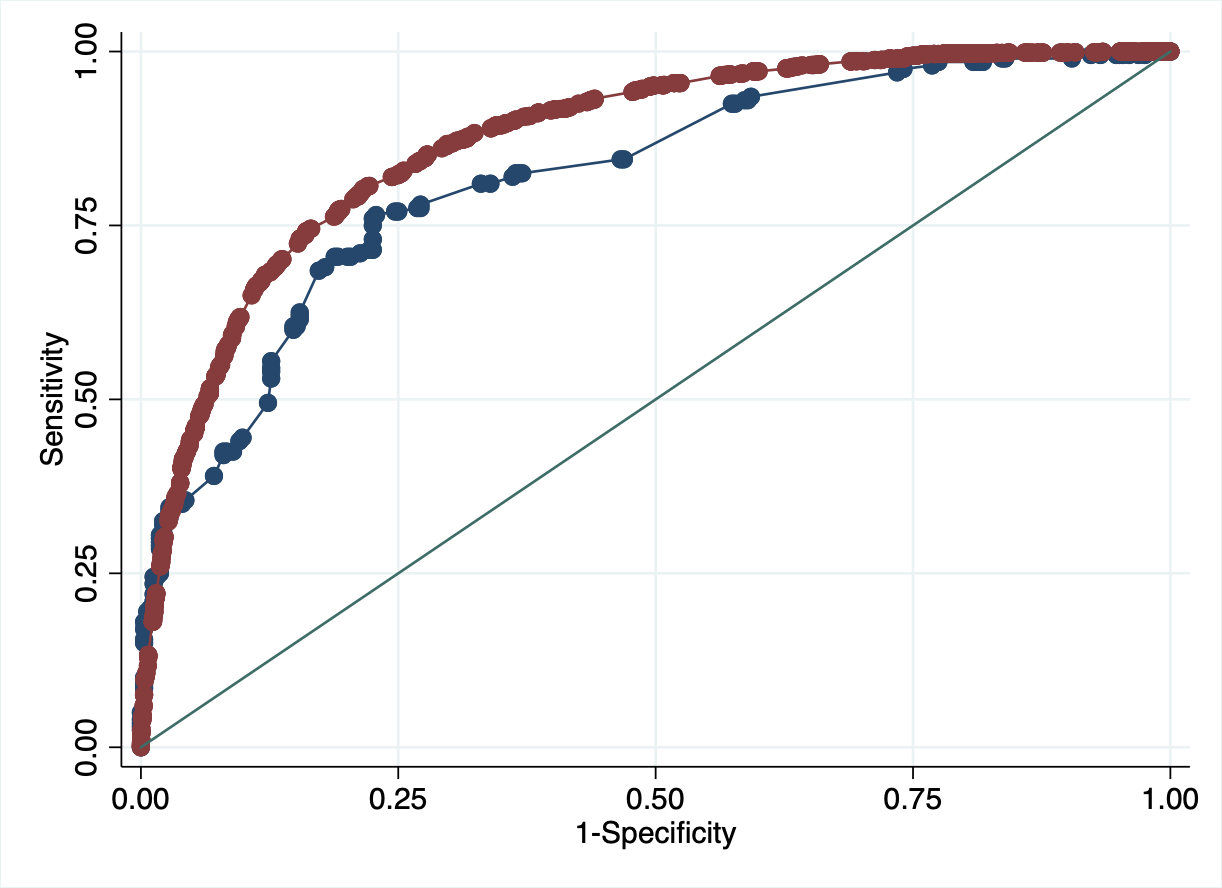
**

**Supplementary figure 1.** ROC curves showing the ability of the 9 SNP T1D GRS to discriminate between T1D and controls in Indians (blue: AUC [95% CI] 0.82 [0.78-0.85]) and Europeans (red: AUC [95% CI] 0.87 [0.86-0.88]), *P*<0.0060.


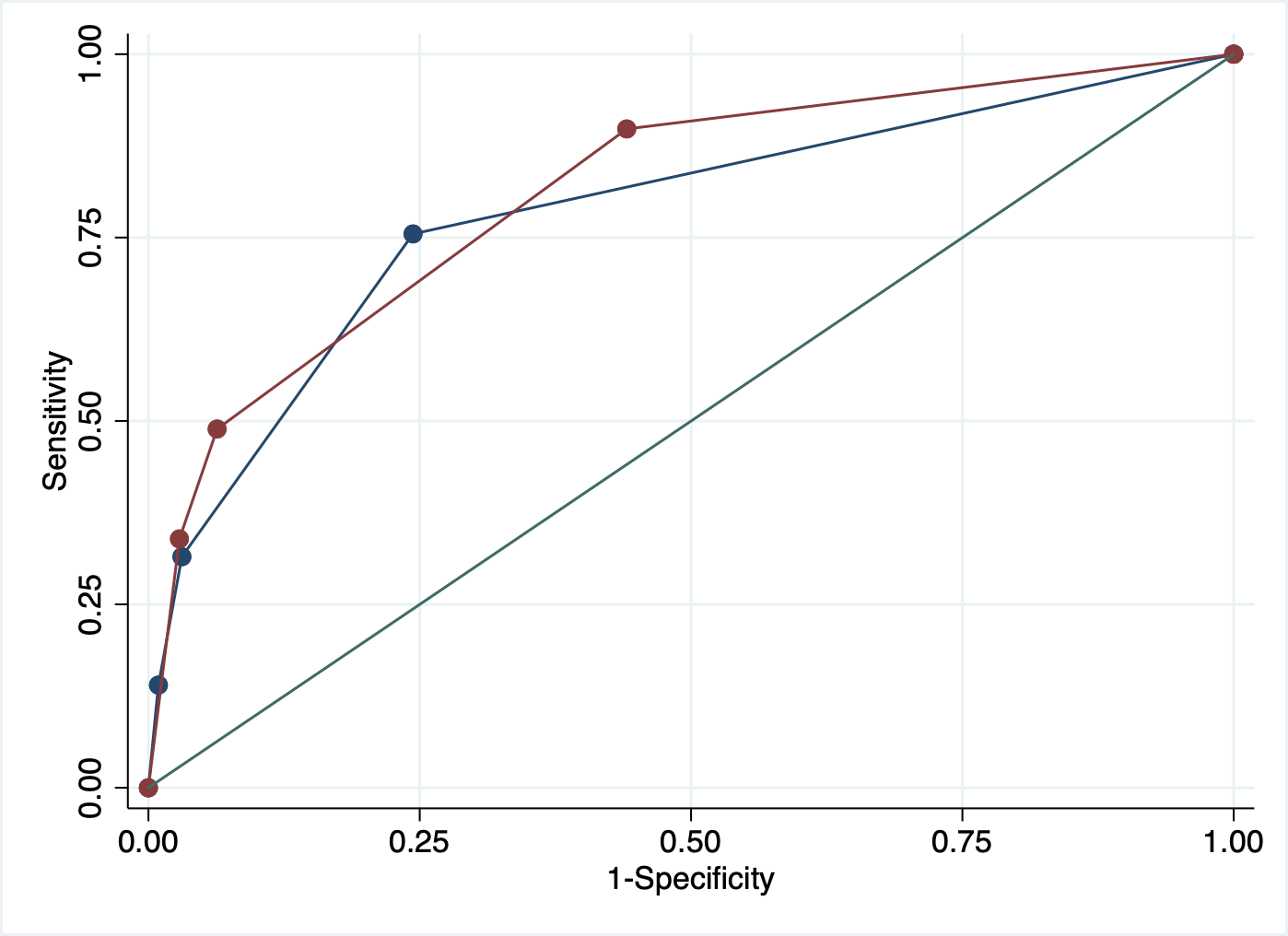


**Supplementary figure 2.** ROC curves showing the ability of imputed HLA DR3/DR4 status to discriminate between T1D and controls in Indians (blue: AUC [95% CI] 0.79 [0.75-0.83]) and Europeans (red: AUC [95% CI] 0.81 [0.80-0.82]), *P*=0.16.

**
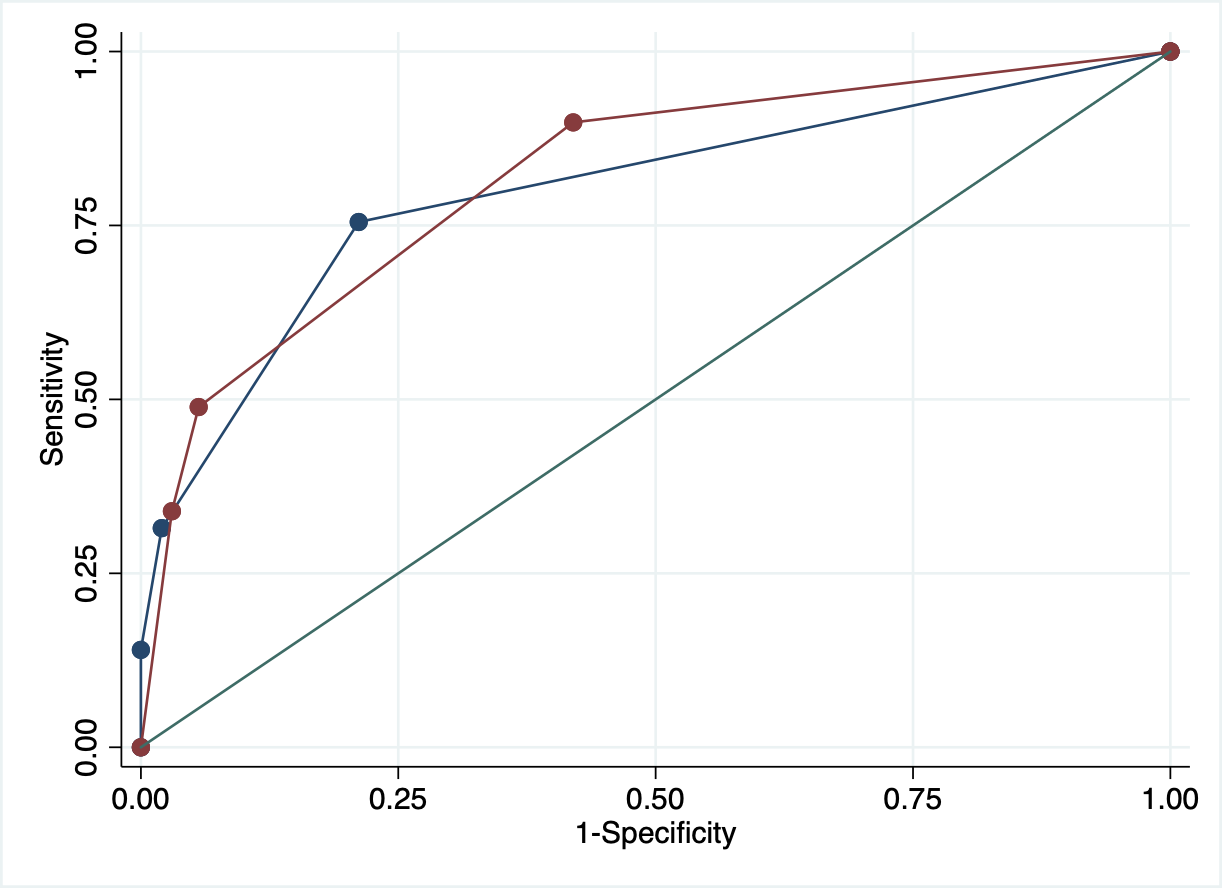
**

**Supplementary figure 3.** ROC curves showing the power of imputed HLA DR3/DR4 status to discriminate T1D from T2D in Indians (blue: AUC [95% CI] 0.78 [0.74-0.82]) and Europeans (red: AUC [95% CI] 0.82 [0.81-0.83]), *P*=0.31.


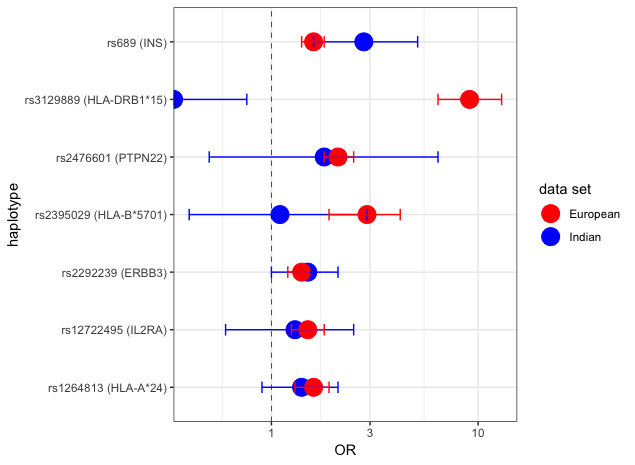


**Supplementary figure 4.** Comparison of odds ratios for non DR3/DR4 variants between Indians (blue circle) and Europeans (red circle) study. Bars show 95% confidence intervals.
